# Annual Ailments

**Published:** 1854-07

**Authors:** 


HALL'S JOURNAL OF HEALTH.

VOL. I.] JULY, 1854. [NO. VII.

### ANNUAL AILMENTS.

Some persons are sick once a year. In some cases, the regularity is such, that on the very same day of each returning year, their "old enemy" makes his unwelcome appearance. These ailments are various; with some, it is an attack of sick head-ache; others have an entire loss of appetite; a third person has some kind of an eruption. Regular annual returns of "biliousness" are very common; a sore leg, a chronic head-ache, or bleeding from the nose or lungs, afflict others. One man has a yearly "sneezing spell," another a most uncomfortable watering of the eyes or nose, while the great mass of people have "The Spring Fever," which was a familiar by-word in our school-boy days, and was a covert way of telling one that he was lazy; for while there was no decided sickness, no special ailment, yet there was such a *vis inertia*, such a power of doing nothing, that an epithet of some kind was needed. On the approach of warm weather, in the month of April, and more decidedly so in May, we are all sensible of a want of usual vigor; an indefinable languor pervades the whole man, mind and body; when we sit down, we feel like staying there; it is really an effort to undertake anything; we drag ourselves along to necessary work; and as for getting up in the morning, we are never ready to do it. We wake soon enough, especially when there is some little yearling to crawl over and manipulate the nose, or explore the eye with a straight finger suddenly converted into a hook, and then drawn out with infinite glee; no gesture, or growl, or impatient turning over, frightens away the little fisherman; in fact he rather likes it; it is real fun to him: then incontinently he makes a grab at Proboscis with his soft, warm, tiny hand, and misses it just enough to let two or three sharp finger-nails "make their mark" for an inch or so in parallel lines; at length the corner of one unwilling eye is opened with the express purpose of seeing in what direction you must send your frown, when you find two of the sweetest little peepers playing upon you so confiding, so loving, so twinkling with gleesomeness, that, pressing the tiny tormentor to your bosom, you smother him with kisses, and are fairly waked up. This is the sweetest alarm-clock in all nature, and the most effectual. As regular as the dawn, too, while it practises—a perseverance worthy of a better cause—than breaking up a summer morning's nap.

We began this journal with the fixed purpose, at least, until we changed our notion, never to admit to its pages a single medicinal recipe. But, inasmuch as there is such a large class of persons who "can't wake up" early in the morning, we are tempted by the hope of accomplishing a great good, to give the following formula:—

AN INFALLIBLE PREVENTIVE OF LATE SLEEPING.

*Take a year old baby—your own is the best—place it in your bed every evening at sixty-one minutes after sun-down, precisely, and retire regularly yourself four hours after.*

This is immeasurely superior to any alarm-clock ever constructed, for the clock has no second, if the first fails, while the baby keeps on alarming, until the waking-up is perfect and complete, and a relapse for that day is impossible.

Perhaps the reader may remember that we were speaking of the "Spring Fever," that universal lassitude which makes us mere automata at the departure of cold weather. But this is not the only symptom; the appetite begins to flag, our meals come before we are ready for them, and we sit down to them unwillingly; if we enjoy the bliss of boarding, we begin to complain of the landlady, or lord, as the case may be, and grumble threats of making a change; that the table is not as good as it used to be, and the old saw about "new brooms," and their performances, refreshes our memories; at length, things begin to take a serious turn; our clothing does not fit, it hangs like a bag; and to quench uncertainty we get on one of Fairbanks best, and find that we have lost in two months about "seven per cent," and pronouncing it a "ruinous rate," we promptly resolve, and with a good deal of determination, too, that we must "*do* something." In this case, the first resolutions are the best, but as we think it over, we come to the conclusion that it would be better to *take* something, and as the calling in of a physician endangers our largest liberty, and he might impose restrictions which might not be agreeable, we resolve upon a patent medicine, and if not sooner advised, or, if having no choice ourselves, we are at a loss to determine what is best, we very wisely go to a druggist and ask him if he has not something that will do us good; of course he has, having at least half a dollar clear interest in every bottle he sells; or it may be, we see an advertisement in one of the papers, reading like the following:—

"CERTIFICATE"—A MODEL OF ITS KIND.

*Dear Doctor:*

I will be one hundred and seventy-five years old next October. For ninety-four years I have been an invalid, unable to move except when stirred with a lever; but a year ago last Thursday, I heard of the Granicular Syrup. I bought a bottle, smelt of the cork, and found myself a new man. I can now run twelve and a-half miles an hour, and throw nineteen double somersets without stopping.

P. S.—A little of your Alicumstoutum Salve applied to a wooden leg, reduced a compound fracture in nineteen minutes, and is now covering the limb with a fresh cuticle of white gum pine bark.

We go at once to the "Patent Medicine Depot," and ask the shopman if the "Granicular" and "Alicumstoutum" are really good, he assures us that according to his best interest and belief, they have cured persons "a great deal worse off" than we are; so to make assurance doubly sure, we purchase a bottle of each, and take a dose of both thrice a day, on the principle that if one medicine cures everything, two medicines will cure all, and more too, and we will not only get well of our present ailments, but all that are to come. Thus it is, the patent medicine men live in up-town palaces, have their beautiful villas on the banks of the Hudson, build splendid stores on Broadway, and drive in unexceptionable equipages; and to make all these go in the same direction as their physic, they head subscription lists, especially the published ones, with their hundreds and their thousands—meanwhile we. their victims, go down to our graves, unsuspecting why or how.

This brings us to our second reminder of "Spring Fever," or rather its termination. We will now take the back track, and discourse of its cause, its cure, and its prevention. Reader! it will save you many a sorrow, many a dollar, and may-be, many a day of glorious life, if you will take heed to our utterance.

We eat about one-third more in winter than in summer, because we not only have to repair the wear and waste of the system, but we eat to keep the body warm, a portion of the food is converted into fuel; we must keep a bodily warmth of ninety or a hundred degrees winter and summer, but it is easy to understand, that, as the thermometer is at forty in winter and eighty in summer, less fuel is required to sustain the natural temperature in warm weather; yet, if in defiance of this, we pile on the fuel; a wreck and ruin is as inevitable as the blowing up of a steam engine, if double the necessary quantity of steam is constantly generated.

For a while after the opening of spring, we have the appetite of winter, and not using our knowledge, we indulge it as extensively; and thus generating more heat than is needed, we soon begin to think "we are feverish," in other words, we are too warm, but instead of making less fire, we begin to tear down the walls of our bodily-house, by taking off our winter clothing, and thus add another cause of disease and death. In a short time, however, nature comes to our aid, and to save us, takes away our appetite; but we, taking this as an evidence of declining health, decide upon one of two things: either to eat without an appetite—which is expressively denominated as "*forcing it down*"—or we decide upon taking a tonic, forgetting that nature can neither be forced nor coaxed with impunity. The effect of eating without an appetite, or forcing an appetite by the use of tonics, is the same, that is, the introduction of more food into the stomach than nature requires, than there is juices to digest it; for, although you may take a tonic which whets the appetite, it does no more; it does not increase the amount of gastric juice, for nature supplies it only in proportion to the needs of the system, and if she gave as much when twenty degrees of heat were required as when sixty were necessary, she would commit a great blunder—this she never does, when unmolested. Then we have more food in the stomach than there are gastric juice for; more wheat than there are mills to grind it, more work than there are workmen to perform. But nature has not "a lazy bone in her," but goes to work to do the best she can; the food is digested, but not thoroughly; it is ground up, but not perfectly; the work is done, but it is badly done; hence an imperfect material for making blood is furnished; and an impure blood, an imperfect blood, is inevitable. Do not many of us recollect the old time custom of taking "sassafras" tea in spring, or some other favorite remedy, with the expressed intention of "purifying the blood?" It is a habit with multitudes to use some kind of medicine in the spring of the year, and beyond all question with present good effect, as they all act in one way essentially, and that is, to remove the surplus from the system. It all amounts to this: we eat in the spring more than we can dispose of, and then take medicine to get rid of it, and all for the transient pleasure enjoyed for the few minutes of each day that it is passing down the throat. But some are "principled," as they term it, against taking physic; but they are not "principled" against the greater harm of eating against the appetite, for by taking the physic they would be consuming something which might destroy others; whereas, by eating a meal without an appetite, they consume—and that to their own injury—that which would save many a famishing creature, man or beast, from starvation. None can read without disgust of a Roman Ruler, who would eat to his full, then take an emetic, that he might eat again, or be saved from the effects of a gorge. Even this person acted more wisely than does he who eats without an appetite, and allows it to remain in him to vitiate the blood and finally destroy the body, but in the slow process of destruction, affording time to transmit to the innocent unborn, a vitiated constitution, to afflict and plague for untold years to come. If the man could but die in the act, as it were, the world would be left the better, for there would be more left to be eaten by the more worthy, and he would not leave his slimy trail behind him, in the person of a child. If he is said to be a real benefactor to his race who makes two blades of grass grow where but one grew before, what ought he to be thought of, who absolutely destroys food each day by forcing it down, which it would take a million blades of grass to reproduce? Reader! do you plead guilty to having eaten without an appetite, to having "forced it down?"

then do it no more, for it is a sin against yourself, against nature, and against all human kind.

We are all familiar with the prevalence of bowel-complaints of all kinds, in the spring of the year, and of their fatal nature, sometimes spreading from house to house, from family to family, from neighborhood to neighborhood, like some infectious or contagious disease, and often, but most erroneously, attributed to the use of fruits, berries, and the like; the cause is one and universal: it is over eating, with its legitimate results, sour stomach, wind, loose bowels, debility, diarrhœa, dysentery, and death. Thus it is, that the more sudden the coming on of spring weather, and the hotter it is, the more sickness there will be, while in the fall of the year, as the weather gets colder, and however suddenly, we begin at once to gain in appetite, in vigor, in flesh and health.

The remedy for spring diseases, by whatever name, is EAT LESS. We do not mean that you shall starve yourself, or that you shall deny yourself whatever you like best, for as a general rule, what you like best, is best for you; you need not abandon the use of tea, or coffee, or meat, or anything else you like, but simply eat less of them. Eat all you did in winter, if you like, but take less in amount. Do not starve yourself, do not reduce the quantity of food to an amount which would scarcely "keep a chicken alive," but make a beginning, by not going to the table at all, unless you feel hungry; for if you once get there, you will begin to taste this and that and the other, by virtue of vinegar, or mustard, or syrup, or cake, or "something nice;" thus a fictitious appetite is waked up, and before you know it, you have eaten a hearty meal, to your own surprise, and perhaps that, or something else, of those at table with you.

The second step towards the effectual prevention of all spring diseases, summer-complaints, and the like, is: diminish the amount of food consumed at each meal by one-fourth of each article, and to be practical, it is necessary to be specific; if you have taken two cups of coffee, or tea, at a meal, take a cup and a-half; if you have taken two biscuits, or slices of bread, take one and a-half; if you have taken two spoonfuls of rice, or hominy, or cracked wheat, or grits, or farina, take one and a-half; if you have taken a certain, or uncertain quantity of meat, diminish it by a quarter, and keep on diminishing in proportion as the weather becomes warmer, until you arrive at the points of safety and health, and they are two:—

1. Until you have no one unpleasant feeling of any kind after your meals.

2. Until you have not eaten so much at one meal, but that when the next comes, you shall feel decidedly hungry.

If these suggestions are attended to in any community, in any spring, the physician in that locality will "book" but twenty-five cents in the dollar. Think, for a moment, how beautiful, and wise, and kind, is nature's mode of procedure in such cases. You may "force" your food for a-while, but at length she goads you on until you loathe its very smell or sight, or even mention. And when some mistaken mother, or sister, or aunt, or granny, prepares you "something nice," and before you are aware of it places it right under your nose, with the assurance that you must take something to keep up your strength, you can only smother your impatience, or hold in your imprecations of left-handed blessings, at the expense of the most heroic efforts. For days and weeks you do nothing but "sit around;" you eat nothing, you loaf and lounge about, more dead than alive, with a countenance nineteen yards long, and so unfeignedly solemn to behold, as to excite the commiseration of the kind-hearted, or the cachination of the healthy.

Supplies being thus effectually cut off, that is, the cause being first removed, nature next proceeds to work off the surplus, as the engineer does unwanted steam; and as soon as this surplus is got rid of, we begin to improve; the appetite, the strength, the health return by slow and safe degrees, and we at length declare we are "as well as ever."

Now, if instead of eating against nature, we would do at once what nature will enevitably compel us to do, sooner or later, that is, lessen supplies, at the very first and faintest intimation of approaching ill, we would, *if done under the counsel of a regular physician*, not lose an hour from our daily avocations, and would be as well as ever at the end of a week, instead of at the end of months, and save, too, all the months of suffering, and making them, as to pleasure or business, the blanks of our existence.

But the uninformed and the poor are "not able to lose time,"

they must work for their daily bread, and the day they cease their toil, has no bread for wife and children at its close; thus it is that many an honest poor man, and many a widowed mother, strive to weather it out, day after day, eating without an appetite, in the mistaken notion that it keeps up their strength, until the system becomes so impregnated with disease, that at last they reluctantly "give up," they find "it's no use;" take to their bed, and but too often, not until all the restorative energies of nature are gone, and never leave until they make it in the grave. It is this vain, this unwise struggle against nature, this doing more than they are able, which places many an industrious wife and mother in an early grave; the almost universal excuse is, they "can't help it," there is "so much to be done." But if they die, isn't it helped then? with your children left to grow up in neglect, or to be brutalized over by some unprincipled, or selfish, or unfeeling, or artful successor? Mother! look on your little ones, and think of this the next time you commit the sin of doing more than you are really able, against the remonstrance of your husband, your mother, your physician, and your own judgment.

According to my observation, there is not much danger of a man's overdoing himself. The first thing the lord of creation does when he gets "out of kelter" is to go to his wife to be "fussed over." A half-and-half sick man is the veriest "co-nanny" in existence. Reader, do you know what that is? It is the synonym of "poor shoat," in the west; perhaps a clearer idea may be given by the word "calf." A half-and-half sick man is a calf, for he makes a great a-do about nothing; he whines, and complains and grumbles, and makes you think he is very sick, and for a long time he refuses to take anything; at length, by dint of persuasion, he agrees to take what you propose, and when you bring it to him, he is out of the notion, and says he can't, and thus he lounges about the house for days together, whereas his wife, *if nothing more had been the matter with her*, would have worked it off, and said nothing about it.

---

There is a gentleman named Standard, now living, at the age of 88, who distinctly remembers hearing the first volley fired in the Revolutionary war, at Lexington, on the 19th of April, 1775. He was then 9 years old.

